# Introducing Spatial Information into Predictive NF-κB Modelling – An Agent-Based Approach

**DOI:** 10.1371/journal.pone.0002367

**Published:** 2008-06-04

**Authors:** Mark Pogson, Mike Holcombe, Rod Smallwood, Eva Qwarnstrom

**Affiliations:** 1 Department of Computer Science, University of Sheffield, Sheffield, United Kingdom; 2 Unit of Cell Biology, Section of Infection Immunity and Inflammation, University of Sheffield, Sheffield, United Kingdom; University of the Western Cape, South Africa

## Abstract

Nature is governed by local interactions among lower-level sub-units, whether at the cell, organ, organism, or colony level. Adaptive system behaviour emerges via these interactions, which integrate the activity of the sub-units. To understand the system level it is necessary to understand the underlying local interactions. Successful models of local interactions at different levels of biological organisation, including epithelial tissue and ant colonies, have demonstrated the benefits of such ‘agent-based’ modelling [1]–[4]. Here we present an agent-based approach to modelling a crucial biological system – the intracellular NF-κB signalling pathway. The pathway is vital to immune response regulation, and is fundamental to basic survival in a range of species [5]–[7]. Alterations in pathway regulation underlie a variety of diseases, including atherosclerosis and arthritis. Our modelling of individual molecules, receptors and genes provides a more comprehensive outline of regulatory network mechanisms than previously possible with equation-based approaches [8]. The method also permits consideration of structural parameters in pathway regulation; here we predict that inhibition of NF-κB is directly affected by actin filaments of the cytoskeleton sequestering excess inhibitors, therefore regulating steady-state and feedback behaviour.

## Introduction

NF-κB is a transcription factor which is central to the regulation of genes involved in inflammatory and immune responses. Activation of NF-κB and its associated pathway of interactions is controlled by inhibitors of NF-κB (IκB) proteins, which sequester the majority of NF-κB in the cytoplasm as complexes by masking their nuclear localisation signals [9]. During activation, IκB is phosphorylated by IκB kinases (IKK), causing its destruction [10], [11]. The newly freed NF-κB is consequently transported into the nucleus, inducing inflammatory genes, including those encoding IκB, thus regulating the pathway through negative feedback [12], [13].

Pathway activation is tightly controlled at multiple levels. Detailed information of the parameters regulating specific steps and their impact on activation is of fundamental importance for understanding the pathway as a whole. Recently, modelling of regulation at the level of the inhibitor has been performed using differential equations, helping to improve understanding of pathway operation and regulation [14], [15]. We aim to take a different approach to modelling the pathway in order to gain a different perspective on its operation, with a greater focus on spatial detail and a more direct comparison with experiment.

Our agent-based model can provide a more complete appreciation of the regulatory mechanisms within the signalling network as a whole, demonstrating predictive behaviour at all steps from initiation at the level of the cell-surface receptor (TIR) to resultant gene regulation. The model extends the capabilities of reaction kinetics and stochastic simulation models by including explicit spatial and structural parameters such as localisation, transport, and complex formation of signalling intermediates, thus relating directly to real time single cell analysis.

Any number (within computational limitations) and distribution of molecules can be modelled, time delays in key processes are properly accounted for, and individual interactions of agents are characterised by stable and well-defined parameters. The model reflects the discrete stochastic nature of interactions, and provides a realistic description of subcellular events.

Computational modelling is a rapidly developing methodology for investigating the organisation of complex biological systems. Such modelling allows in virtuo experiments to complement the in vitro and in vivo methods that are already well-established in biology. The flexible, intuitive and extensible nature of agent-based modelling makes it well-suited to modelling biological systems. It requires the identification of an appropriate level to model (in our case the cell) and the entities to be modelled (here individual molecules); the complete system is derived from considering interactions of the individual components with the environment and neighbouring components, and the behaviour of the complete system is an emergent property. The behaviour of an individual component is determined by the dynamics of its internal characteristics (state), its physical location, and its relationships with those components around it (communication). Modern computing power and experience of modelling complex systems composed of many interacting autonomous parts have provided the foundation for this approach to understanding complex biological systems.

## Results

In the model, molecular agents diffuse through the cell, binding and dissociating from other molecules, receptors and cell structures in accord with signals they send and receive from surrounding agents. Every agent is represented by a complete computational model – the communicating stream X-machine – which provides an intuitive and rigorous basis to model the functional behaviour of systems in a flexible and extensible manner [16], [17]. An important feature is the memory of each agent's X-machine, which contains its physical location, meaning that the number of states required to model the system is manageably small.

**Figure 1:**
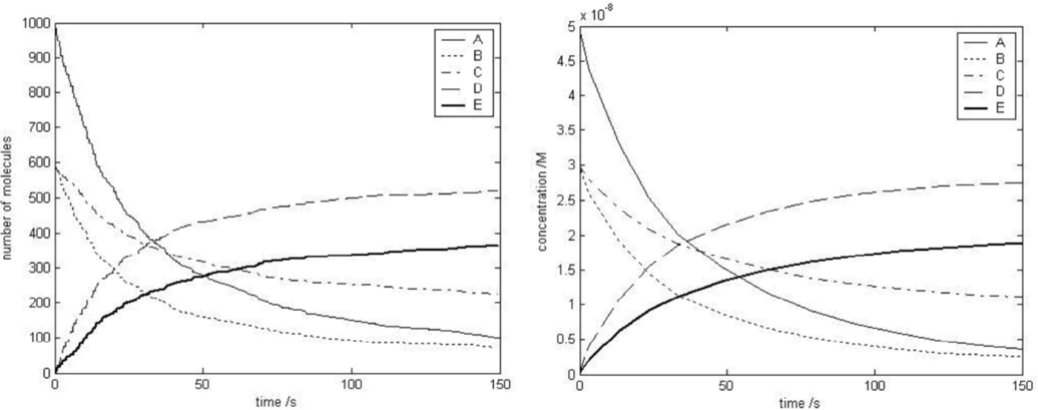
Agent interactions. Agreement of agent-based model of chemical interactions with a model based on reaction kinetics ODEs. Reaction A+B−>D, A+C−>E. Left: agent model results. Right: corresponding ODE results.

It is essential that the agents are both biologically plausible as entities and that their behaviour is based on experimental measurements. In the model, as in reality, molecular interactions are local events that depend only on the position and current state of the molecules involved, where the state of a molecule is whether or not it is already bound. The physics of a molecule is modelled according to specific agent-based characteristics, including which types of interaction are possible. If two molecules may interact according to the rules, they must satisfy criteria on their state and proximity, derived from standard rate constants [18]. If interaction occurs, the state of each agent changes to a ‘bound’ state, which can be reversed through random thermal separation. The model agrees with reaction kinetics in homogenous situations (Fig. 1).

**Figure 2:**
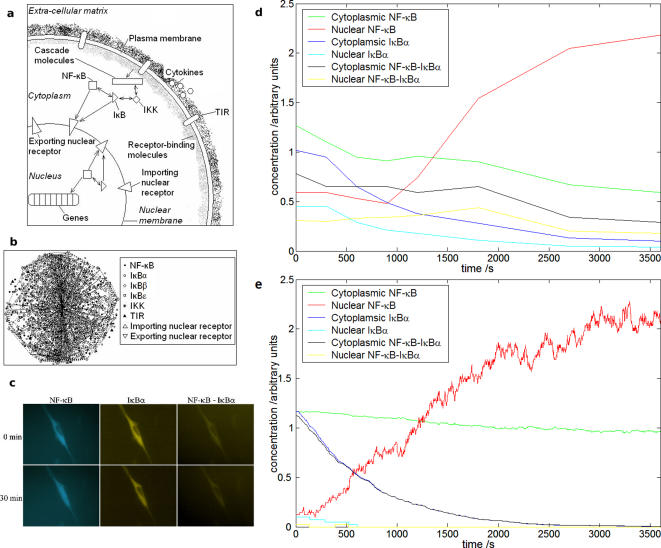
Formulation and validation of the agent-based model. (a) Simplified diagram of the principal pathway agents in the model. Each agent can exist in a number of states. (b) Three-dimensional visualisation of the positions of agents in the model at a moment in time. The cell is scaled down to reduce computation, containing in the order of 1000 agents. Concentrations of molecules are based on biological data. The genes that NF-κB can activate are placed randomly along a line at the centre of the nucleus. (c) Images of single cells co-transfected with ECFPrelA and IκBαEYFP. Prior to stimulation, both components are located in the cytoplasm (top row). Following pathway activation, NF-κB translocates to the nucleus (left) whilst IκBα (centre) and NF-κB-IκBα complex levels (right) fall (bottom row). (d) Quantitation of single cell data as in (c). (e) Model results following TIR activation over the same time period. Results are for a single cell, and demonstrate fundamental similarities with experiment. Standard error 5.4% for nuclear concentration in model.

TIR mediated activation of the pathway is considered. Each NF-κB, IκB and IκB-kinase (IKK) molecule is an individual agent, as are the importing and exporting nuclear and cell surface receptors (Fig. 2a). Soluble extracellular agonists are not modelled as agents but treated as a whole chemical entity whose fluctuating local concentrations at the cell surface must rise above a certain level to initiate signal transduction in nearby receptor agents. Similarly, in relation to the cytoplasmic TIR domain, the local concentrations of certain molecules in the cytoplasm must be above a defined level in the vicinity of an active receptor to complete the process. Following this, a temporary agent with an internal time delay is created to account for the cascade that triggers the IκB kinases (IKKs). At the final steps of activation, an analogous temporary agent method is used to account for the translation of IκB.

The agents are contained within a spherical cell consisting of a cytoplasm and concentric spherical nucleus (Fig. 2b). Space is continuous and time is discrete in the model. The rules that govern agent movements incorporate cell structure by defining spatial boundaries. This allows investigation of the impact of cell shape and biomechanical effects [19].

The model is in good agreement with biological data obtained by real time single cell analysis (Fig 2c–e). Continuous monitoring of signal transduction events in live cells was performed using GFP-tagged regulatory intermediates and confocal microscopy [5]–[7]. Simultaneous observations of the NF-κB subunit relA and IκBα were carried out using cyan (ECFP) and yellow (EYFP) variants of GFP respectively, as previously [7]. Complex-formation was assessed by measuring fluorescence resonance energy transfer (FRET), with ECFPrelA the donor and IκBαEYFP the acceptor.

**Figure 3:**
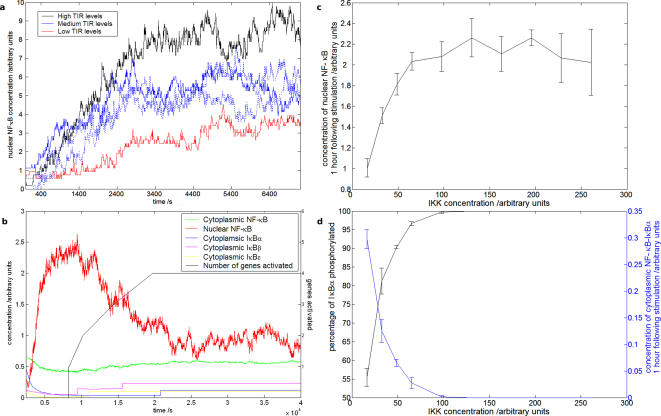
Investigating the pathway in further detail with the model. (a) Positive correlation between saturated TIR levels and pathway activity at the level of NF-κB nuclear translocation. Dashed lines indicate control runs. (b) Extended kinetic analysis following stimulation. System returns to non-stimulated state following activation. (c) Effect of IκB kinase (IKK) on pathway regulation. Increasing IKK levels demonstrates concentration dependent activation and saturation of activity at key regulatory steps, effect on nuclear translocation of NF-κB. (d) Effects on IκBα phosphorylation and NF-κB-IκBα complex levels. All error bars show standard error of the mean.

The robustness of the agent-based model, in contrast to the instability often found in ODE models, allows it to be used to assess effects across the pathway as a whole, such as to determine the impact of alterations at the level of the cell surface receptor on downstream signalling events and gene regulation (Fig. 3a), here demonstrating an expected positive correlation between TIR levels and nuclear translocation of NF-κB.

The model is not as restricted as experiment in the duration or detail that can be observed. Results obtained over an extended period demonstrate the expected system switch-off (Fig. 3b), in agreement with induction of negative feedback mechanisms following activation. In addition, the model demonstrates distinct regulation by IκB isoforms, and control of gene activity.

**Figure 4:**
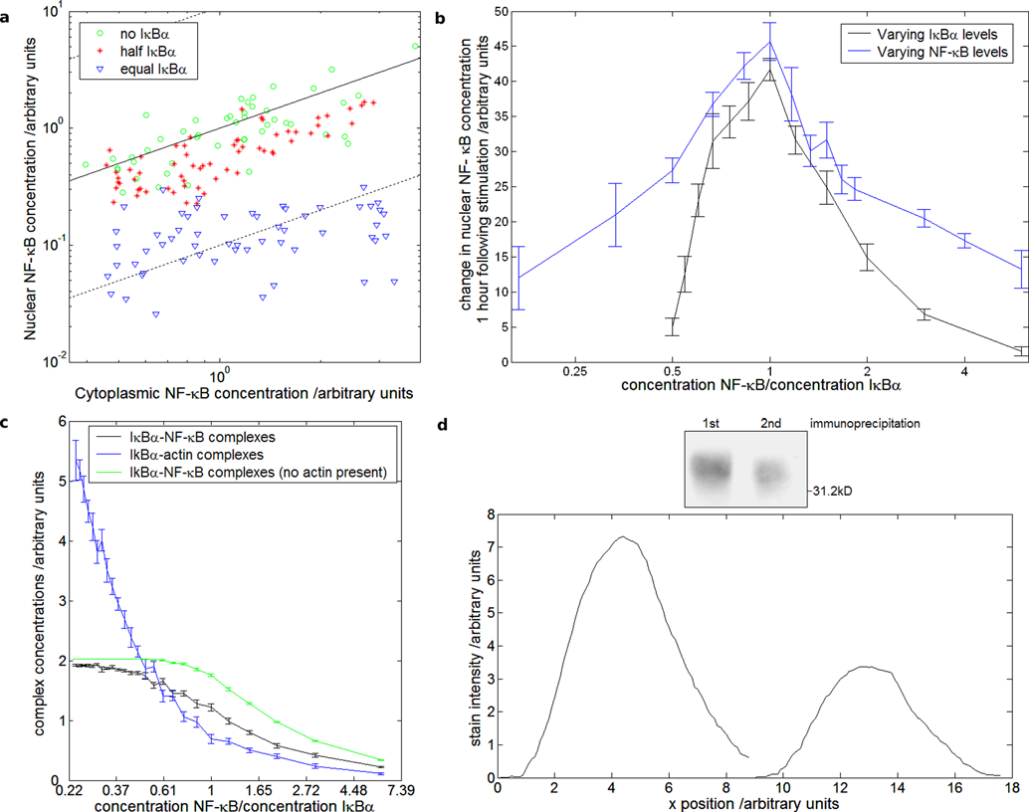
(a) Relative nuclear and cytoplasmic concentrations of NF-κB with varying IκBα levels at equilibrium. Uninhibited, the cytoplasmic∶nuclear concentration of NF-κB is approximately 1∶1 (solid diagonal line); increasing inhibitor levels to equal those of NF-κB results in a 10∶1 cytoplasmic∶nuclear concentration of NF-κB (lower dashed diagonal line), in close agreement with experiment. (b) Effects of changing NF-κB to IκBα ratios on responses stimulation by varying IκBα levels at constant NF-κB (black line) or varying NF-κB levels at constant IκBα (blue line). (c) NF-κB-IκBα and actin-IκBκ levels at equilibrium with changing relative NF-κB∶IκBα concentrations (NF-κB concentration constant). With actin present, the maximum NF-κB-IκBα complex level is reached at around 1∶3 NF-κB∶IκB concentration (black line), with the excess IκB bound to actin (blue line). The same maximum is reached at around 1∶1 concentration in the absence of actin (green line). (d) Top: Western analysis of IκBα following crosslinking and immuniprecipitation using an anti-actin antibody (p1, left lane) and in a sequential IκBα immunoprecipitation of remaining supernatant (p2, right lane). Bottom: Scanning of gels as above. Quantitation of three independent experiments, using NIH image, showed an average ratio of actin-bound IκBα to free IκBα of 2∶1+/−10%. One representative experiment is shown.

The model permits investigation of various pathway features, such as the effects of changing levels of signalling proteins at specific regulatory steps. The model displays a narrow range of IKK levels within which subsequent pathway activity is affected, measured at the level of nuclear transport of NF-κB (Fig. 3c) and IκBα phosphorylation and complex dissociation (Fig. 3d). The impact of relative levels of NF-κB and IκBα on cellular localisation is demonstrated in Fig. 4a, showing a concentration dependent decrease in relative nuclear NF-κB levels with enhanced inhibitor levels, reflecting complex formation and cytoplasmic retention, mimicking the resting state, as in previous biological experiments [5]–[7]. Further analysis demonstrates, as expected, a maximal increase in NF-κB nuclear translocation at a 1∶1 NF-κB∶IκB ratio (Fig. 4b). This, together with a more potent effect of changing inhibitor levels, is in agreement with the significance of sequestering excess inhibitor in pathway control.

The spatial detail of the model permits explicit modelling of structural components of the cell such as the cytoskeleton, which is partly composed of actin filaments, to which IκB can bind [20]. NF-κB-IκB complex formation at equilibrium was assessed in the presence of actin-IκBα interactions (Fig. 4c). With actin present, maximal NF-κB-IκB complex formation was achieved at a 1∶3 NF-κB∶IκBα ratio, in agreement with levels measured in live cells during optimal activation. Without actin, the maximum is reached at a 1∶1 concentration, corresponding to data in Fig. 4a. The model therefore predicts a key role for IκBα-actin interactions possibly related to sustaining optimal pathway regulation by adjusting NF-κB-IκB complex formation at the steady-state, and controlling negative feedback following activation. Biological experiment using sequential immunoprecipitation supports these findings, demonstrating a ratio of actin-bound to free IκBα of 2∶1±10% in unstimulated cells; the results for one of three of these experiments is presented in Fig. 4d.

## Discussion

The agent-based modelling paradigm illustrated here provides a useful technique for understanding many aspects of biological systems. It is a suitable model for cellular regulatory events such as the NF-κB pathway, providing a clear and intuitive mechanism to determine and explore the key features of the system.

The model provides a biologically validated quantitative result that would likely not be found by existing models, not least due to the different model specification: prior to stimulation, ODE models tend to allow inhibitor concentrations to reach equilibrium via transcription, with an initial concentration of zero in the system. Given the lack of actin or other means for sequestration, this results in a non-physiological concentration of approximately 1∶1 rather than 3∶1 IkB∶NF-kB.

The detailed mathematical structure of the model should also allow the use of automated reasoning techniques such as model checking to properly understand the system in all possible circumstances within which it operates [21]. This is an important capability that has not yet been exploited in biological modelling.

## Materials and Methods

### Computational Model

The coding of the model is of the form: 1. define parameters for chemical reactions and cell dimensions; 2. assign molecular positions; 3. iterate each time step, calculating agent movements and potential interactions, and updating agent states as appropriate. The sequence of reaction calculations is randomised at each time step to prevent bias towards any type of interaction. The model is scaled down to improve computation time; this is justified in light of experiments to determine its effect, and is comparable to standard practice in stochastic differential equation modelling [22].

Actin filaments were assumed to have the same attraction to IκB molecules as to NF-κB, with 8000 filaments included in the model cell as an estimate for actual values, though variations around these values did not have a significant effect on system results.

### Biological experiment

#### Cell culture

Hela cells were propagated at 37°C in 5% CO_2_ and maintained in DMEM (Gibco) supplemented with 10% foetal calf serum (Gibco).

#### Confocal microscopy

To determine effects on IκBα turnover, cells grown in 8-well chamber-slides (1.0×10^4^–1.0×10^5^ cells/well) were transfected with pIκBα-EGFP (0.2 µg/well), using the calciumphosphate method, as described [5]–[7]. Twenty-four hours after transfection, cells were stimulated with IL-1β (10^−9^ M). During activation, until one hour, continuous monitoring of the fusion proteins was carried out using confocal microscopy (Molecular Dynamics Multiprobe 2010, 488 nM, 40× Plan Apo oil immersion, NA 1.4, 50-µm aperture, optical sect 0.54 µm, laser power 10 mW, band selection of 488 mn, PMT of 750, and varying attenuation to maintain pixel density below 200 which is within the linear range of the instrument), as previously [7]. To quantitate the cytoplasmic and the nuclear fusion protein levels, transfected cells were scanned horizontally through the nucleus. Images were converted to 8bit TIFF format, analyzed using NIH image and relative fluorescence calculated by measuring the mean intensity of representative areas of nucleus and cytoplasm, and dividing by 2.41 to be consistent with previous results obtained with PMT at 666 V [5], [6]. The relationship between fluorescence intensity and fusion protein expression levels was determined by Western analyses and compared with a series of standards, as described in our earlier publications [5], [7].

#### FRET analysis

To determine the level of complex formation, cells were co-transfected with IκBα-ECFP (0.4 µg/well) and EYFP-relA (0.4 µg/well). Images of ECFP, EYFP and FRET were obtained through a 60× Plan Apo oil immersion objective (NA 1.4) using a 12 bit Hamamatsu Digital Camera C4742-95 driven by OpenLab software (Improvision). The following filter sets (Omega Optical) were used: XF114 for ECFP (440DF21 excitation, 455DRLP dichroic, 480DF30 emission), XF104 for EYFP (500DF25 excitation, 525DRLP dichroic, 545DF35 emission) and XF88 for FRET (440DF21 excitation, 455DRLP dichroic, 545DF35 emission). Relative fluorescence was calculated by determining the intensity per pixel for the various emission settings, by measuring the mean intensity of representative areas of nucleus or cytoplasm and normalised by dividing by the attenuation, as above. All images were background corrected and the FRET images were further processed by subtracting overspill of ECFP (50.7%) and EYFP (30.4%), and fluorescence calculated as above.

#### Cross linking and Immunoprecipitation

Cells grown in a 10 cm dish (10^6^) were transfected with IκBα-EGFP using the calcium phosphate method, as previously described [5]. Twenty-four hours after transfection, cells were cross-linked with 5 mM DSS (Pierce) in PBS pH 8.0 for 30 minutes at room temperature. The solution was quenched with 1 M TrisHCl pH 7.5 to a final concentration of 10 mM and left for 15 minutes. Cross-linked cell lysate was prepared using a RIPA buffer kit (Santa Cruz) as per the manufacturer's instructions. Supernatant lysate (S1) was pre-cleared with Protein A/G PLUS-Agarose Immunoprecipitation Reagent (Santa Cruz) and incubated at 4°C for 30 minutes with appropriate IgG (Santa Cruz). One ml of the lysate was incubated at 4°C overnight with rabbit polyclonal anti-Actin antibody (Santa Cruz, sc-1616) and A/G agarose beads and cleared by centrifugation (13,000 rpm). The pellet (P1) was reserved for Western analysis and the supernatant lysate (S1) was used in a second immunoprecipitation, and incubated with an anti-IκBα mouse monoclonal antibody (Santa Cruz sc-1643; 4°C,30 min). Extracts recovered from the pellet after a second centrifugation (13,000 rpm) (P2) were analysed by Western blotting.

#### SDS-PAGE and western analysis

Immunoprecipitates were boiled in 1× Laemelli sample buffer and separated on SDS-PAGE (10%) and transferred onto PVDF membrane (Hybond-P, Amerham). Following blocking (5% milk powder in TBS/Tween ,1 hr, rt) membranes were incubated for with a rabbit polyclonal anti-IκBα antibody (Santa Cruz sc-371, 1 µg/ml, 2 hrs, rt). Following washes (TBS/Tween and TBS), membranes were incubated with HRP conjugated anti-rabbit IgG (1∶2000, Santa Cruz, 1 hour) and subsequently with Luminol Reagent (Santa Cruz,1 min) before visualisation by ECL (Amersham Biosciences). Controls included incubation with second antibody alone, and the level of IgG was used as loading control.
